# The efficacy of herbal medicines on the length of stay and negative conversion time/rate outcomes in patients with COVID-19: a systematic review

**DOI:** 10.3389/fphar.2024.1383359

**Published:** 2024-05-30

**Authors:** Irma Rahayu Latarissa, Anna Meiliana, Ida Paulina Sormin, Erizal Sugiono, Nasrul Wathoni, Melisa Intan Barliana, Keri Lestari

**Affiliations:** ^1^ Department of Pharmacology and Clinical Pharmacy, Faculty of Pharmacy, Universitas Padjadjaran, Sumedang, West Java, Indonesia; ^2^ Prodia Clinical Laboratory, Jakarta, Indonesia; ^3^ Faculty of Pharmacy, University of 17 August 1945 Jakarta, Jakarta, Indonesia; ^4^ Prodia Diacro Laboratory, Jakarta, Indonesia; ^5^ Department of Pharmaceutics and Pharmaceutical Technology, Faculty of Pharmacy, Universitas Padjadjaran, Sumedang, West Java, Indonesia; ^6^ Department of Biological Pharmacy, Faculty of Pharmacy, Universitas Padjadjaran, Sumedang, West Java, Indonesia; ^7^ Center of Excellence for Pharmaceutical Care Innovation, Universitas Padjadjaran, Sumedang, West Java, Indonesia

**Keywords:** herbal medicines, randomized controlled trial, clinical trial, antiviral, antiinflammatory, immunomodulatory

## Abstract

**Introduction:**

In recent years, diverse initiatives have been carried out to control the COVID-19 pandemic, ranging from measures restricting social activities to analyzing drugs and vaccines. Studies on herbal medicines are also increasingly conducted in various countries as an adjuvant therapy or supplement. Therefore, this systematic review aimed to investigate the efficacy of herbal medicines analyzed from various countries through clinical trials with the randomized controlled trial method. The outcomes of Length of Stay (LOS), Negative Conversion Time (NCT), and Negative Conversion Rate (NCR) were the main focus.

**Methods:**

An extensive review of literature spanning from 2019 to 2023 was carried out using well-known databases including PubMed, Scopus, and Cochrane. The search included relevant keywords such as “randomized controlled trial,” “COVID-19,” and “herbal medicine.”

**Results:**

A total of 8 articles were part of the inclusion criteria with outcomes of LOS, NCT, and NCR. In terms of LOS outcomes, all types of herbal medicines showed significant results, such as Persian Medicine Herbal (PM Herbal), Persian Barley Water (PBW), Jingyin Granules (JY granules), Reduning Injection, and *Phyllanthus emblica* (Amla). However, only JY granules showed significant results in NCR outcome, while JY granules and Reduning Injection showed significant results in reducing NCT.

**Conclusion:**

These findings enrich our understanding of the potential benefits of herbal medicines in influencing LOS, NCR and NCT parameters in COVID-19 patients. Herbal medicines worked to treat COVID-19 through antiviral, anti-inflammatory, and immunomodulatory mechanisms.

## 1 Introduction

The COVID-19 pandemic, caused by the SARS-CoV-2 virus is a tremendous global challenge in the field of health (Filip et al., 2022). Over the past few years, various efforts have been made to reduce the pandemic, from policies related to social activity restrictions to studies on drugs and vaccines (Lazarus et al., 2022). Even though vaccination has been an important milestone in controlling the spread of the virus, the development of effective treatments is equally important to address the clinical impact (Davis et al., 2020; Machado et al., 2022). Various repurposing drugs have been studied regarding the efficacy and safety against COVID-19 (Babaei et al., 2021; Latarissa et al., 2023). Research on drugs for COVID-19 has made significant progress. However, there are still some challenges. For instance, Ritonavir has several drug interactions that require special evaluation before use (Igho-Osagie et al., 2023). In addition, the high financial cost of purchasing vaccines (Light and Lexchin, 2021). Therefore, there is a need to find more affordable alternatives that have a lorcwer risk of drug interactions. This calls for further research into the use of herbal medicine as a possible effective and economical alternative to deal with this pandemic (Liu et al., 2022).

Herbal medicines, with their long history of use in traditional medicine, offer the potential to contribute to symptom relief, accelerate recovery, and strengthen the immune system. While no herbal preparation has yet been consistently shown to be effective in treating COVID-19, recent studies have shown some promising candidates as potential adjunctive or alternative therapies (Al-kuraishy et al., 2022; Komariah et al., 2023; Kumar et al., 2023; Onyeaghala et al., 2023). This medicine has a long history across different cultures, and some plants have been recognized to possess antiviral, anti-inflammatory, and immunomodulatory properties relevant in the context of viral infections (Nugraha et al., 2020). Herbal medicines that have anti-inflammatory potential can help reduce inflammation in the respiratory tract and other organs due to coronavirus infection, through decreasing inflammatory indicators such as interleukin (IL)-6, erythrocyte sedimentation rate (ESR), and C-reactive protein (CRP) (Zeng et al., 2020). Meanwhile, immunomodulatory properties found in herbal medicines can enhance the immune system’s response to infection, thus helping the body fight the virus (Das, 2022). In terms of antivirals, some herbal medicines show potential antiviral activity against various types of viruses, including the coronavirus (Jamal, 2022; Benzie and Wachtel-Galor, 2011) (19).

Evaluation of clinical trials of herbal medicines includes aspects of safety and efficacy, as well as specific parameters related to the characteristics of the disease. Some of the aspects evaluated are the ability of herbal medicines to relieve symptoms, reduce the severity of the disease, and accelerate patient recovery. In addition, this study also needs to pay attention to the potential of reducing the level of viral replication, inhibiting inflammation, and enhancing the patient’s immune response. An important parameter is the length of stay (LOS), which reflects the efficacy of the treatment in reducing hospital burden and accelerating patient recovery. Reduced length of hospitalization can be a positive indicator that herbal medicines have the potential to resolve symptoms and reduce the severity of the disease (Ang et al., 2022). In addition, Negative Conversion Rate (NCR) and Negative Conversion Time (NCT), including the length of time required for patients to achieve negative laboratory test results are also critical parameters in evaluating the effectiveness of herbal medicines. The faster the negative conversion occurs, the better the herbal medicines are considered to overcome the viral infection and accelerate the healing process (Jeon et al., 2022).

This systematic review aims to investigate the effects of herbal medicines on decreasing patient LOS, NCR, and NCT in the context of COVID-19. Evaluation of these parameters provides a more comprehensive understanding of the efficacy and potential of herbal medicines as part of the treatment strategy. The results of this systematic review have great scientific importance and significant relevance in the context of pandemic management. First, in terms of LOS outcomes, it can provide a deeper understanding of the effectiveness of herbal medicines in reducing patient LOS to optimize the use of limited health resources and accelerate patient recovery. Second, in terms of NCR outcomes, this review can provide a comprehensive analysis of how many patients are cured by using a particular herbal medicines to guide health practitioners in choosing the most effective therapy. Third, in terms of NCT outcomes, this review can provide insight into the potential of herbal medicines in accelerating the negative conversion time of patients infected with COVID-19. Meanwhile, the novelty of this review is that it focuses on specific outcomes so that readers can infer results based on these outcomes. Thus, this systematic review will be a very important tool for healthcare practitioners, researchers, and policymakers in making informed decisions and leading to a more holistic understanding of the role of herbal medicines in responding to the COVID-19 pandemic.

## 2 Methods

### 2.1 Article selection

**FIGURE 1:**
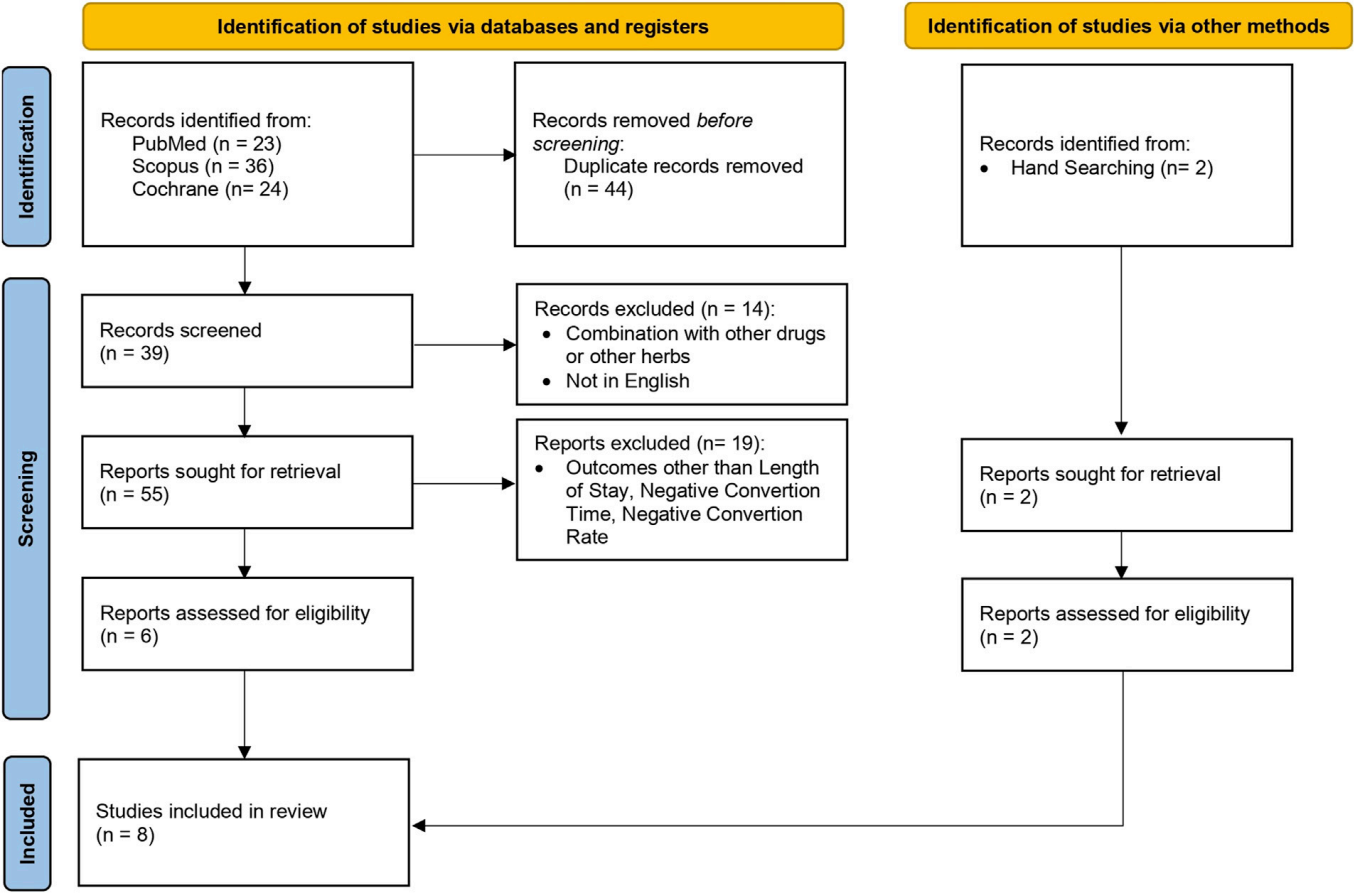
PRISMA flowchart: the study selection.

This systematic review included full original articles obtained through PubMed, Scopus, and Cochrane. During this search, original studies meeting the defined criteria were identified. Boolean operators such as “OR” and “AND” were used to expand the exploration within each concept and refine the results, respectively. Medical Subject Headings (MeSH) terms were also applied and the detailed search terms are outlined in Table 1. Furthermore, the original study published from 2019 to 2023 was used regarding clinical trials of herbal medicines with a randomized controlled method in English and the study subject was human. There were also study articles that did not clearly state the intervention, such as book chapters, article abstracts only, conference reports, reviews, posters, and discussion results. The articles identified for inclusion were screened and the findings were discussed to reach consensus. The procedure for selecting study articles is shown in Figure 1.

**TABLE 1:** Search strategy in database searching.

Database	Search terms
PubMed	("COVID-19"[MeSH Terms] OR "SARS-CoV-2"[MeSH Terms] OR "COVID-19"[Title/Abstract]) AND ("herbal medicine"[Title/Abstract] OR "herbal plants"[Title/Abstract] OR "herbs"[MeSH Terms] AND "randomized controlled trial"[Publication Type]
Scopus	(COVID-19 OR " SARS-CoV-2") AND (herbal medicine OR herbal plants OR herbs) AND ((randomized AND controlled AND trial) OR (randomized AND controlled AND trial))
Cochrane	((MeSH descriptor: [COVID-19] explode all trees) OR SARS-CoV-2) OR (MeSH descriptor: [herbal medicine] explode all trees) OR (MeSH descriptor: [herbal plants] explode all trees) OR (MeSH descriptor: [herbs] explode all trees) OR ((herbal medicine):ti,ab,kw) OR ((herbal plants):ti,ab,kw) OR ((herbs):ti,ab,kw))

### 2.2 Subject characteristics

Subjects involved in the studies that were included in this systematic review were patients with positive reverse transcription polymerase chain reaction (RT-PCR) test for COVID-19, who had mild or moderate symptoms. Inclusion criteria included patients >18 years of age, and there were no gender restrictions. Patients with severe symptoms were excluded from this study.

### 2.3 Outcomes

The studies included in this analysis evaluated the efficacy of herbal medicines based on specific outcomes such as LOS, NCR and NCT. The LOS for inpatients with COVID-19 refers to the duration of time that individuals diagnosed with COVID-19 spend in hospital care, from admission to discharge (Wen et al., 2022). NCT for inpatients with COVID-19 refers to the duration it takes for a patient to transition from testing positive for the virus to testing negative (Bob-Manuel et al., 2021). Meanwhile, NCR refers to the proportion of patients who transition from testing positive for the virus to testing negative within a certain timeframe (Hu et al., 2020).

### 2.4 Data extraction and risk of bias

IRL and NW screened all relevant articles by evaluating their titles and abstracts. The full articles deemed suitable were reviewed and approved by IRL. Data extraction was conducted utilizing a review matrix created with Microsoft Excel and included author names, the active compound, country, sample size, patient characteristics, design study and outcomes (LOS, NCT and NCR). The risk of bias was conducted utilizing the Jadad score, which focused on three main guidelines for evaluating articles: randomization, double blinding, and the description of withdrawals and dropouts. Five questions need to be answered within a 10-min timeframe: Was the study described as randomized (using terms like random, randomly, or randomization)? Was the method for generating the randomization sequence adequately described and suitable? Was the study identified as double-blind? Was the method of double blinding adequately described and appropriate? Was there a description provided for withdrawals and dropouts?

The Jadad score was chosen for the systematic review because of its advantage in providing a simple yet systematic assessment of the methodological quality of clinical studies. The tool focuses on three main elements: randomization, double blinding, and missing data reports, which are considered important indicators in evaluating the quality of a study. With a clear and standardized approach, the Jadad score allows researchers to directly compare the methodological quality of different studies, making it possible to make a more objective assessment of the evidence. In addition, the simplicity of the tool also facilitates its implementation in systematic analyses involving a large number of studies. (Jadad et al., 1996; Olivo et al., 2008).

## 3 Results

### 3.1 Outcomes on length of stay

**FIGURE 2:**
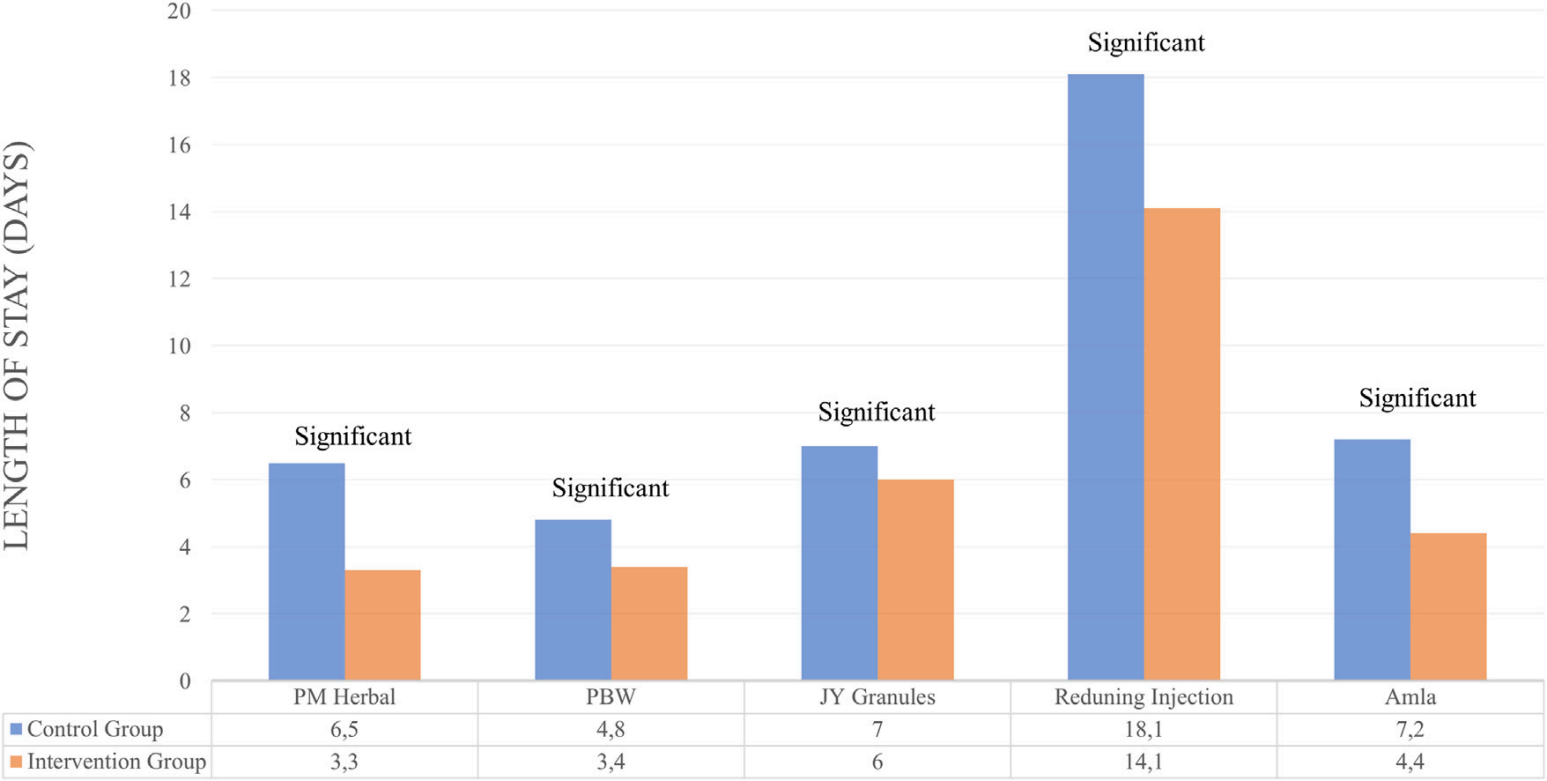
Efficacy of herbal medicines on LOS outcomes.

The 5 herbs in the inclusion criteria gave positive results where the LOS in the test group was better than the control and showed significant differences (Karimi et al., 2021; Xu et al., 2021; Tavakoli et al., 2022; Varnasseri et al., 2022; Chen et al., 2023). Meanwhile, the standard care used for each herbal medicine was adjusted to the value of each country. The duration of administration ranged from 7 days for Persian Medicine Herbal (PM Herbal) and Jingyin Granules (JY granules), 10 days for *Phyllanthus emblica* (Amla), and 14 days for Persian Barley Water (PBW) and Reduning Injection. A summary of the results for the efficacy of herbal medicines on LOS outcomes is provided in Table 2 and Figure 2.

**TABLE 2:** Efficacy of herbal medicines on LOS outcomes.

Herbal medicines	Active compound	Country	Sample size	Patient characteristics	Design study		Outcomes on length of stay
					Control group	Intervention group	
Persian Medicine Herbal (PM Herbal) (Karimi et al., 2021)	Capsule 1 (*Rheum palmatum* L.; *Glycyrrhiza glabra* L.; *Punica granatum* L.) Capsule 2 (*Nigella sativa* L.) Herbal decoctation (*Matricaria chamomilla* L.; *Zataria multiflora* Boiss.; *Glycyrrhiza glabra* L.; *Ziziphus jujuba* mill.; *Ficus carica* L.; *Urtica dioica* L.; *Althaea officinalis* L.; *Nepeta bracteate* Benth.	Iran	358 patients	Positive on RT-PCR, had pneumonia confirmed by chest imaging and had an oxygen saturation ≤93% on room air	Standard care for 7 days (n = 174 patients)	Standard care for 7 days + herbal remedies (polyherbal decoction every 8 h and two herbal capsules every 12 h) (n = 184 patients)	Significantly decreased LOS (*p* < 0.001, hazard ratio = 0.3). Patients in the test group had a shorter LOS than those in the control group (3.291 days vs. 6.468 days)
Persian Barley Water (PBW) (Tavakoli et al., 2022)	*Hordeum vulgare*	Iran	100 patients	COVID-19 patients with moderate disease severity at least 18 years	Standard care (n = 50 patients)	Standard care + PBW 250 mL daily for 2 weeks (n = 50 patients)	The test group significantly decreased the LOS by 4.5 days (95% CI: −7.22, −1.79 days) in comparison to the control group. The test and control groups stayed at the hospital for 3.41 (95% CI: 2.59, 4.23) and 4.84 (95% CI: 4.09, 5.58) days, respectively.
Jingyin Granules (JY granules) (Chen et al., 2023)	*Schizonepeta tenuifolia* Brig., *Lonicera japonica, Taraxacum officinale, Isatis indigotica* Fort., *Ilex purpurea* Hassk., *Houttuynia cordata, Arctium lappa, Saposhnikovia divaricata, Glycyrrhiza glabra*	China	791 patients	Patients infected with SARS-CoV-2 who had mild symptoms and no imaging signs of pneumonia, age ≥18 years	Standard care + placebo (n = 423 patients)	Standard care + JY granules for 7 days (n = 368 patients)	The LOS was shortened by 1 day in the test group compared with control (6.0 days vs. 7.0 days, *p* < 0.001)
Reduning Injection (Xu et al., 2021)	*Artemisia annua, Lonicera japonica* Thunb, and *Gardenia jasminoides* Ellis	China	157 patients	Diagnosed with COVID-19, one or more symptoms among fever, cough and fatigue, ≥18 years old	Standard care (n = 80 patients)	Standard care + Reduning Injection 20 mL/day for 14 days (n = 77 patients)	The test group had a shorter LOS (14.1 vs. 18.1 days, *p* < 0.001)
*Phyllanthus emblica* (Amla) (Varnasseri et al., 2022)	*Phyllanthus emblica*	Iran	61 patients	Age over 18 years, a positive RT-PCR test for COVID-19, pulmonary inclusion on chest imaging, hospitalization	Standard care (hydroxychloroquine sulfate + lopinavir/ritonavir) (n = 30 patients)	Standard care (hydroxychloroquine sulfate + lopinavir/ritonavir) + 2 g of the sachet powder of Amla or 100 cc Amla tea per day for 10 days (n = 61 patients)	The mean of LOS in the test group (4.44 days) was significantly shorter than in the control group (7.18 days, *p* < 0.001)

### 3.2 Outcomes on NCT/NCR

Several studies use NCR and NCT outcomes to determine the efficacy of herbal medicines. However, some only use one of the NCR or NCT outcomes. For Lianhuaqingwen Capsules (LH capsules), JY granules and Kovir capsule used NCR and NCT outcomes simultaneously. For LH capsules, the results showed no significant differences in the control and test groups for NCR and NCT parameters (Hu et al., 2021). Similarly, in the Kovir capsule, where the results showed no significant difference in the NCR or NCT parameters (Loc et al., 2022). JY granules showed good results, significantly increasing NCR and also decreasing the average of NCT (Chen et al., 2023).

**FIGURE 3:**
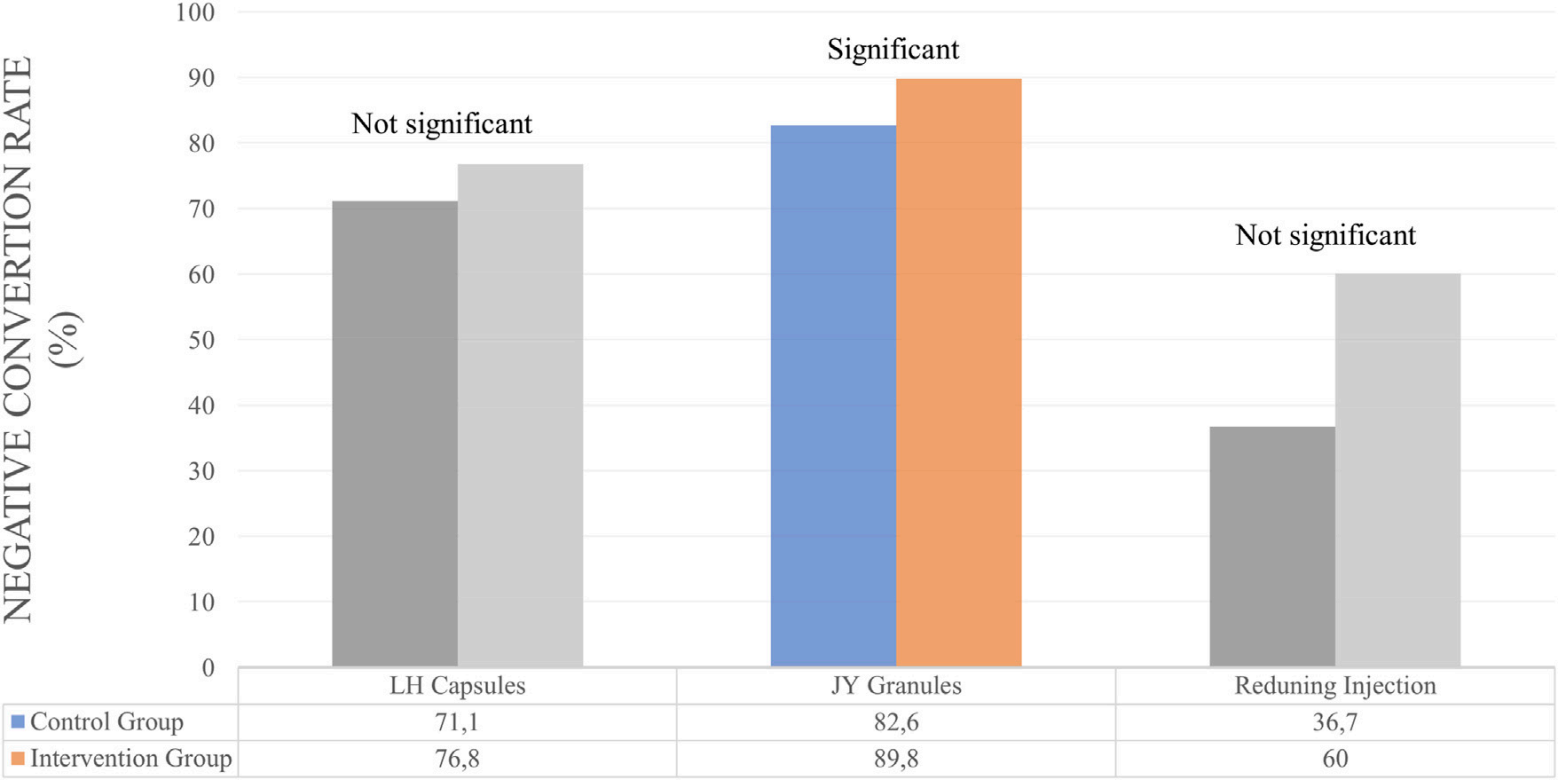
Efficacy of herbal medicines on NCR outcomes.

**FIGURE 4:**
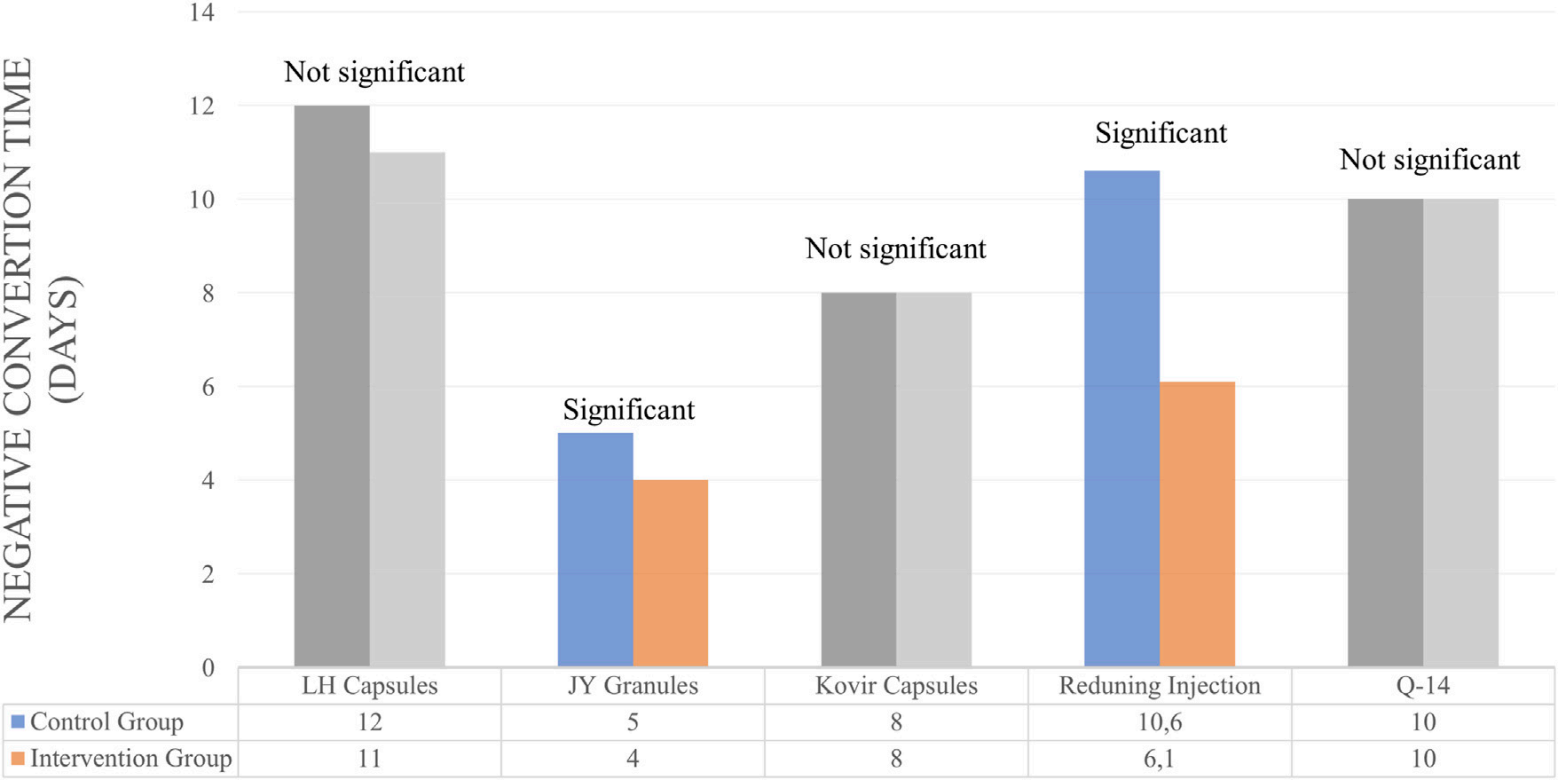
Efficacy of herbal medicines on NCT outcomes.

Studies that only use NCR parameters to determine the efficacy of therapy is Amla. There was no significant difference between the control and test groups (Varnasseri et al., 2022). Herbs that use NCT as a parameter of therapeutic outcome are Reduning Injection and Hua Shi Bai Du Granule (Q-14 granule). Reduning Injection shows good therapeutic results where the herb can significantly reduce NCT (Xu et al., 2021). Meanwhile, the herbal Q-14 granule did not significantly differ in NCT parameters (Liu et al., 2021). The duration of herbal administration for these patients is 7, 10, and 14 days. A summary of the results for the efficacy of herbal medicines on NCT and NCR outcomes is provided in Table 3 and Figures 3, 4.

**TABLE 3:** Efficacy of herbal medicines on NCR and NCT outcomes.

Herbal medicines	Active compound	Country	Sample size	Patient characteristics	Design study		Outcomes on NCR	Outcomes on NCT
					Control group	Intervention group		
Lianhuaqingwen Capsules (LH capsules) (Hu et al., 2021)	*Forsythia suspensa*, *Lonicera japonica*, *Ephedra sinica*, *Isatis indigotica*, *Pogostemon cablin*, *Rheum palmatum*, *Glycyrrhiza uralensis*, *Dryopteris crassirhizoma*, *Rhodiola crenulata*, *Houttuynia cordata*, *Prunus sibirica*	China	284 patients	Laboratory-confirmed cases with COVID-19, being symptomatic, patients aged 18 years or greater of either sex.	Standard care (n = 142 patients)	Standard care +4 capsules LH, thrice daily for 14 days (n = 142 patients)	No significant difference in the NCR between the test group and control group (FAS: 76.8% vs. 71.1%, mean difference: 5.6%, 95%CI: −4.6%–15.7%, *p* = 0.279; PPS: 77.0% vs. 71.2%, mean difference: 5.8%, 95%CI: −4.5%–15.9%, *p* = 0.273)	No significant difference in the median NCT between the test group and control group (FAS: 11.0 vs. 12.0 days, HR: 1.21, 95%CI: 0.92–1.59; PPS: 11.0 vs. 12.0 days, HR = 1.21, 95%CI: 0.92–1.59; both *p* = 0.151)
JY granules (Chen et al., 2023)	*Schizonepeta tenuifolia* Brig., *Lonicera japonica, Taraxacum officinale, Isatis indigotica* Fort., *Ilex purpurea* Hassk., *Houttuynia cordata, Arctium lappa, Saposhnikovia divaricata, Glycyrrhiza glabra*	China	791 patients	Patients infected with SARS-CoV-2 who had mild symptoms and no imaging signs of pneumonia, age ≥18 years	Standard care + placebo (n = 423 patients)	Standard care + Jingyin granules for 7 days (n = 368 patients)	A significantly greater increase in NCR than control was shown in the test group (89.8% [380/423] vs. 82.6% [304/368], *p* = 0.003)	The median NCT of the test group was decreased significantly compared with that of the control group (4.0 [3.0, 6.0] days vs. 5.0 [4.0, 7.0] days, *p* < 0.001)
Kovir capsule (Loc et al., 2022)	*Bupleurum chinense* DC., *Poria cocos* (Schw.) Wolf., *Codonopsis pilosula* (Franch.) Nannf., *Peucedanum decursivum* Maxim, *Glycyrrhiza glabra* L., *Platycodon grandiflorum* (Jacq.) A. DC., *Ligusticum wallichii* Franch., *Citrus aurantium* L., *Notopterygium incisum* Ting ex H. T. Chang, *Angelica pubescens* Maxim., *Zingiber officinale* Rose., *Mentha arvensis* L.	Vietnam	68 patients	Patients aged from 18 to 65 years who were PCR-confirmed with SARS-CoV-2 and had the mild disease	Standard care + placebo up to 14 days (n = 32 patients)	Standard care +3 tablets Kovir 3 times a day up to 14 days (n = 34 patients)		No significant difference. Viral clearance time was similar in both groups with a median of 8 days
*Phyllanthus emblica* (Amla) (Varnasseri et al., 2022)	*Phyllanthus emblica*	Iran	61 patients	Age over 18 years, a positive RT-PCR test for COVID-19, pulmonary inclusion on chest imaging	Standard care (hydroxychloroquine sulfate + Lopinavir/ritonavir) (n = 30 patients)	Standard care (hydroxychloroquine sulfate + lopinavir/ritonavir) + 2 g of the sachet powder of Amla or 100 cc Amla tea per day for 10 days (n = 61 patients)	There was no significant difference between the control and intervention groups (36,7% vs. 60%, *p* = 0.07)	
Reduning Injection (Xu et al., 2021)	*Artemisia annua, Lonicera japonica* Thunb, and *Gardenia jasminoides* Ellis	China	157 patients	Diagnosed with COVID-19, one or more symptoms among fever, cough and fatigue, ≥18 years old	Standard care (n = 80 patients)	Standard care + Reduning Injection 20 mL/day for 14 days (n = 77 patients)		In the FAS, compared with the control group, the test group had a shorter NCT, significantly (146.5 vs. 255.5 h, *p* < 0.001)
Hua Shi Bai Du Granule (Q-14 granule) (Liu et al., 2021)	*Ephedra sinica* Stapf., *Prunus armeniaca* L., *Glycyrrhiza glabra* L., *Pogostemon cablin* Benth., *Magnolia officinalis* Rehde, *Atractylodes lancea*, *Glycyrrhiza uralensis* Fisch, *Pinellia ternata*, *Poria cocos, Rheum palmatum* L., *Astragalus mongholicus* Bunge, *Descurainia sophia, Paeonia lactiflora* Pall.	China	149 patients	Compliance with the diagnostic criteria for general type COVID-19 in the NHC NATCM-China guidelines (version 6.0); 18 ≤ aged ≤75 years	Standard care for 14 days (n = 78 patients)	Standard care + Q-14 granule 10 g twice daily for 14 days (n = 71 patients)		There was no statistical significance in the NCT between the test group and control group (Full analysis set: Median [interquartile range]: 10.00 [9.00–11.00] vs. 10.00 [9.00–11.00]; Mean rank: 67.92 vs. 81.44; *p* = 0.051)

### 3.3 Quality assessment of the included studies

Quality assessment of the included studies is a critical step in evaluating the reliability and validity of research results. One of the commonly used methods to conduct such an assessment is by using the Jadad score. The Jadad score is a tool used to assess the methodological quality of clinical studies. The total value of the Jadad score ranges from 0 to 5, where higher values indicate higher methodological quality (Jadad et al., 1996). There were no complete scores in the reviewed studies. Two studies had the highest score of 4 (Loc et al., 2022; Varnasseri et al., 2022). The studies fully described the randomization and blinding methods used but did not explain the description of withdrawal/dropout. Meanwhile, a study by Chen et al., 2019 had the lowest Jadad score of 1, because it did not use randomization and blinding methods but a prospective cohort study (Chen et al., 2023). However, we still included this study in the review because it fell within the inclusion criteria. Two other studies did not use blinding methods and did not explain the description of withdrawal/dropout so they had a Jadad score of 2 (Karimi et al., 2021; Tavakoli et al., 2022). Three studies used a single-blind method and did not explain the description of withdrawal/dropout and therefore had a Jadad score of 3 (Hu et al., 2021; Liu et al., 2021; Xu et al., 2021). Table 4 provides an overview of the quality assessment by Jadad score.

**TABLE 4:** Quality assessment by Jadad score.

Author	Randomization	Description of randomization	Double-blind method	Description of the blinding method	Description of withdrawal/dropout	Total score
Karimi et al., 2021	1	1	0	0	0	2
Tavakoli et al., 2022	1	1	0	0	0	2
Chen et al., 2023	0	0	0	0	1	1
Xu et al., 2021	1	1	0	1	0	3
Varnasseri et al., 2022	1	1	1	1	0	4
Hu et al., 2021	1	1	0	0	1	3
Loc et al., 2021	1	1	1	1	0	4
Liu et al., 2021	1	1	0	1	0	3

## 4 Discussion

### 4.1 Mechanism of herbal medicines for COVID-19

**FIGURE 5:**
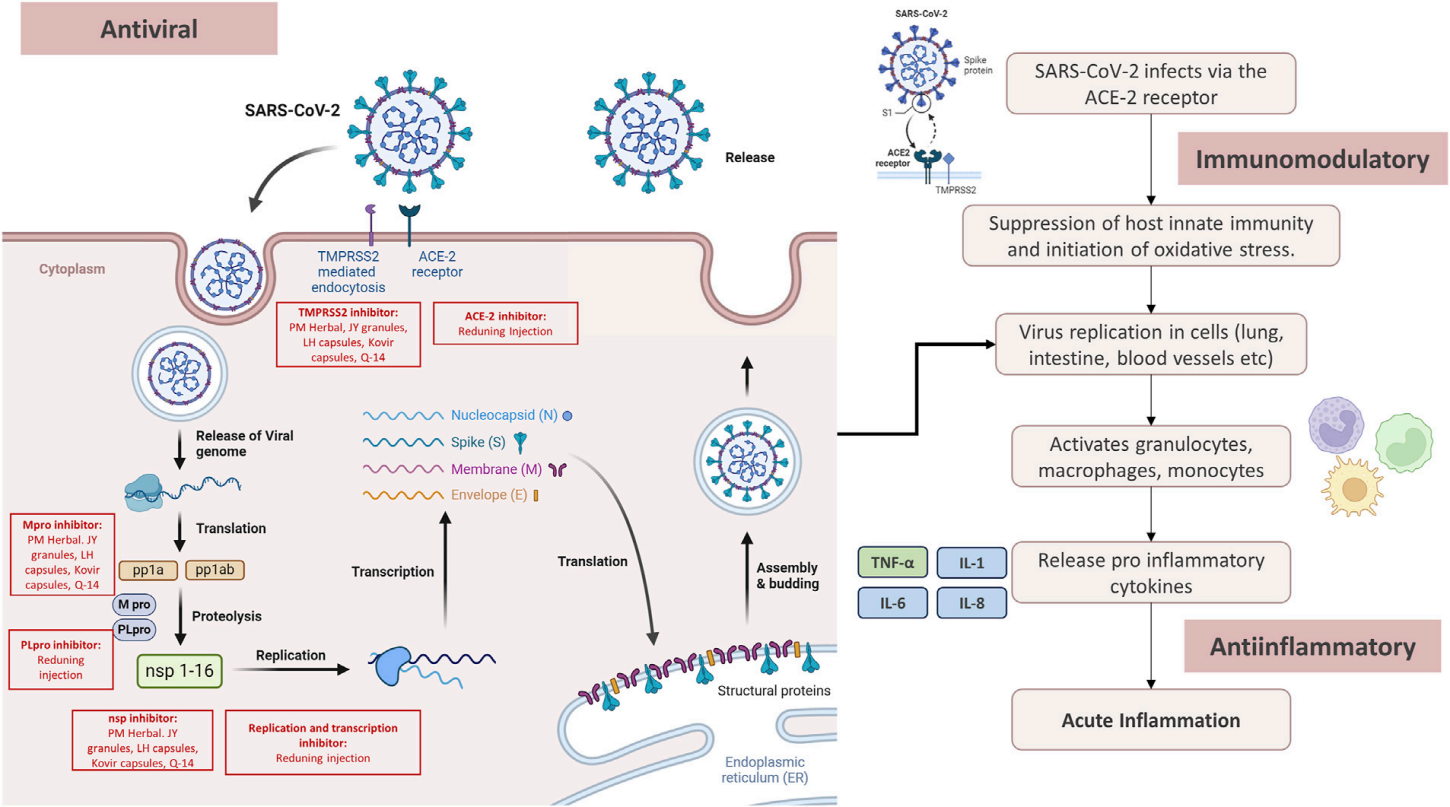
The mechanism of herbal medicines for COVID-19.

The pandemic has triggered intensive studies to obtain effective therapies for the disease. In addition, herbal medicines have also become the focus of attention as an alternative treatment (Demeke et al., 2021). Various medicinal plants have been investigated for their potential antiviral, anti-inflammatory, and immunomodulatory effects assisting the treatment of COVID-19 (Latarissa et al., 2021; Lim et al., 2021). The mechanism of herbal medicines for COVID-19 related to antiviral, anti-inflammatory and immunomodulatory is shown in Figure 5.

#### 4.1.1 Antiviral

Several herbal medicines have shown antiviral capabilities that can reduce the replication of the SARS-CoV-2. The major SARS-CoV-2 protease (Mpro), also known as 3CL protease, plays a key role in processing viral polyproteins generated from SARS-CoV-2 Ribonucleic acid (RNA) translation. This stage has important significance in the virus replication cycle (Zhang and Wang, 2002). Glycyrrhizin, as one of the components in PM Herbal, JY granules, LH capsules, Kovir capsule, and Q-14 granule can reduce the replication of SARS-CoV-2 by inhibiting the main protease Mpro (van de Sand et al., 2021). In addition to inhibiting Mpro, glycyrrhizin can also reduce the human transmembrane serine protease (TMPRSS2) responsible for cleaving the SARS-CoV-2 spike protein and facilitating viral penetration into host cells (Hoffmann et al., 2020; van de Sand et al., 2021). The triterpenoid saponin A3 and glycyrrhizic acid found in *Glycyrrhiza glabra* show effectiveness in inhibiting SARS-CoV-2. This is achieved by targeting nsp7 and the receptor-binding domain of the spike protein (Yi et al., 2021). Antiviral activity is also shown by *Artemisia annua* which is one of the ingredients of Reduning Injection. *Artemisia annua* can inhibit SARS-CoV-2 attachment, membrane fusion and internalization into host cells, and reduce viral replication and transcription processes (Amini et al., 2022).

Molecular docking analyses showed that the components present in Reduning Injection possess the capability to spontaneously bind to papain-like protease (PLpro), Mpro, and angiotensin-converting enzyme-2 (ACE-2). This interaction serves to reduce SARS-CoV-2 from entering the body and replicating (Jia et al., 2021). Antiviral activity is also shown by compounds contained in Amla such as chlorogenic acid, quercitrin, and myricetin. The three compounds can inhibit the main viral protein n-CoV-2 after an *in silico* study (Chikhale et al., 2021).

#### 4.1.2 Anti-inflammatory

COVID-19 is a multifaceted systemic illness, and individuals with moderate and severe cases often experience increased inflammation (Baranovskii et al., 2021; Casale et al., 2023). Cytochrome P450 1A (CYP1A) and Cytochrome P450 3A (CYP3A) are important in the generation of certain essential inflammatory factors, including the oxidative metabolites of arachidonic acid. Strong inhibition of CYP1A and CYP3A holds promise for eliciting anti-inflammatory effects (Tallima and El Ridi, 2018). The three plant extracts present in JY granules show anti-inflammatory properties by deactivating CYP3A. Specifically, *Schizonepeta tenuifolia* Brig. reversibly inhibits CYP3A-catalyzed testosterone 6β-hydroxylation, while the inhibition of *Glycyrrhiza glabra* and folium *Ilex purpurea* Hassk. depends on time, dose, and Nicotinamide Adenine Dinucleotide Phosphate (NADPH) (Li et al., 2017; Zhang et al., 2022).

The potent docking capability of quercetin shows that the compound may effectively lower Interleukin-6 (IL-6) levels during inflammatory episodes. This suggests a potential therapeutic approach for addressing COVID-19 (Luo et al., 2022). In addition, β-glycyrrhizic acid acts as a potent anti-inflammatory compound. This regulates inflammation by inhibiting the accumulation of glucocorticoids and the production of reactive oxygen species (ROS) by neutrophils, which are significant mediators of tissue inflammation (Mittal et al., 2014).

Recent studies have shown that β-glucan in PBW can decrease systemic inflammation. This is achieved by reducing the production of leukocyte superoxide and tumor necrosis factor alpha (TNFα), as well as dampening the stimulation of interferon gene expression (Arcidiacono et al., 2019). Shanshan et al., 2019 showed that Reduning Injection had anti-inflammatory activity by inhibiting the overexpression of mitogen-activated protein kinase (MAPK), protein kinase C (PKC), and p65 nuclear factor-κB affecting cytokine levels in COVID-19 patients (Jia et al., 2021).

#### 4.1.3 Immunomodulatory

Compounds with immunomodulatory activity have an important role with a good protective effect against SARS-CoV-2 by preventing the occurrence of cytokine storms (Al-Hajeri et al., 2022). Some of the compounds in herbal medicines have immunomodulatory activity improving the condition of COVID-19 patients. The effects of chamomile heteropolysaccharides found in PM Herbal have immune-stimulating effects on erythrocytes, activation of immune regulatory cells in peripheral blood, and increased sensitivity of helper cells (Gupta et al., 2010). *Punica granatum* L. also has immunomodulatory effects such as the growth stimulant effect of polysaccharides isolated from pomegranate on lymphocytes (Joseph et al., 2012). In addition, the plant is also effective in overcoming respiratory problems to improve the symptoms of COVID-19 (Aziz et al., 2016). Amla also has an immunomodulatory effect for repeated respiratory infections in humans. The extract acts as an adaptogen and enhances immunity through several mechanisms such as increased activity of interleukin-2 (IL-2), NK (natural killer) cells, Antibody-Dependent Cellular Cytotoxicity (ADCC), and Interferon-gamma (IFN-γ) production and inhibits apoptosis (Sai Ram et al., 2002; Sreeramulu and Raghunath, 2010; Antiretrovir and Mamidala, 2012). The active ingredients in LH capsules such as quercetin, luteolin oxalin, and kaempferol have effects as immunomodulators by targeting MAPK (Wang et al., 2021a). This can decrease the release of inflammatory mediators and lung tissue damage due to inflammation (Xu et al., 2008) [NO_PRINTED_FORM] In addition, LH capsules regulate lung immunity (Ding et al., 2017), considering that infections are related to immunity (Xu et al., 2008). Kovir capsule, an herbal originating from Vietnam has an immunostimulatory effect on SARS-CoV-2 *in vitro* to improve the immune system (Pham et al., 2023). This is because the Kovir capsule contains bioactive molecules such as flavonoids, alkaloids, flavanol glycosides, and withaferin with various biological activities to improve health in various aspects (Padhy, 2020).

### 4.2 Outcomes on length of stay

LOS is a parameter often used in clinical trials to determine the efficacy of a drug, and COVID-19 is no exception. Understanding the prognosis can be facilitated by examining the LOS (Alimohamadi et al., 2022). The 5 herbs showed significant differences in reducing LOS in COVID-19 patients. The main mechanisms are antiviral, anti-inflammatory, and immunomodulatory. Other mechanisms to accelerate the healing of patients which are seen from LOS parameters used as an antifever, bronchodilator, and antiasthma features, strengthen the body, and invigorate the lungs (Karimi et al., 2021). These mechanisms support providing improvements in the clinical condition of patients, specifically in cases seen from LOS parameters.

PM Herbal is a system of medicine in the world that dates back 7,000 years with formulations that are beneficial for several viral and bacterial infections and respiratory diseases (Soleymani and Zargaran, 2018; Walsh, 2021). The results of the PM Herbal clinical trial to fight COVID-19 were evidenced by a significant difference in the decrease in LOS for the intervention group and the control group (3,291 days vs. 6,468 days) (Karimi et al., 2021). This is also supported by research showing that the compounds contained in PM Herbal are proven to fight several viruses such as influenza virus, Echovirus type 11, adenovirus, respiratory syncytial virus and coronavirus (Balzarini et al., 1992; Utsunomiya et al., 1997; Liu et al., 2004; Kumaki et al., 2011; Lazreg et al., 2011; Karimi et al., 2020).

PBW is a herb originating from Iran and has long been used as an antitussive, for flu, bronchitis, body pain, fever, and other respiratory symptoms (Kapadia, 2006; De Natale and Pollio, 2007). These effects result from the combination of vitamins, dietary fibers, unsaturated fatty acids, mineral elements, and antioxidants found in PBW (Jo et al., 2020). Its good efficacy against SARS-CoV-2 was also proven by an *in vitro* study where the ricin-based peptide from PBW was able to inhibit Mpro with the half-maximal inhibitory concentration (IC50) of 0.52 nM (Kashyap et al., 2022). Mpro is a cysteine protease that plays an important role in viral RNA replication and transcription (Liu et al., 2020). This *in vitro* study is in line with the results of clinical trials that can reduce patient LOS (Tavakoli et al., 2022).

The JY granules comprise various components that exhibit inhibitory effects on SARS-CoV-2. For instance, Radix glycyrrhizae is included to directly suppress SARS-CoV-2 by acting on the IL-6/signal transducers and activators of the transduction-3 (STAT3) pathway (Luo et al., 2022). In addition, Flos lonicera and its effective ingredients can also reduce the main protease activity of the new coronavirus (Gu et al., 2022). JY granules is an antiviral herbal product that has been used for more than 40 years in China to treat various respiratory problems such as fever, sore throat and cough (Wang et al., 2020). JY granules is known to have antiviral activity that has been proven *in vitro* and *in vivo* against the influenza virus (Wang et al., 2021b).

The antiviral activity of Reduning Injection, specifically for treating pneumonia, is also a potential treatment for COVID-19. Reduning Injection is used for indications of nausea, colds, fever, cough, yellow sputum, and upper respiratory tract infections (Zhang et al., 2013; Cao et al., 2020; Chen et al., 2020; Xie et al., 2020). Several studies have also proven that Ieduning Injection has effects as antipyretic, antiviral, and anti-inflammatory (Cao et al., 2020; Xie et al., 2020). Besides pneumonia, the activity of Reduning Injection against viruses is also proven by its efficacy against H1N1 A influenza (Chen et al., 2020).

The antiviral effect of Amla has been investigated to treat several diseases caused by viruses such as herpes simplex and those causing disorders in the reproductive and respiratory system (Xiang et al., 2011; Arjin et al., 2020). In addition, Amla also has therapeutic effects such as analgesic, antipyretic, anti-spasmolytic, expectorant, and antitussive. This can reduce the symptoms of patients affected by COVID-19 (Perianayagam et al., 2004; Krishnaveni and Mirunalini, 2010; Goel et al., 2014). In addition, Amla also showed antiviral potential against coxsackie virus B3 (CVB3) at concentrations of 7.8 μg/mL, 11.0 μg/mL, and 21.8 μg/mL. These results indicate that the highly oxygenated bisabolene sesquiterpenoid glycoside phyllaemblicins compounds found in Amla have potential against the Hepatitis B virus (Lv et al., 2015). The compound glochicoccinoside D which was also obtained from Amla has antiviral potential against influenza A virus strain H3N2 and hand, foot, mouth virus (enterovirus) EV71 (Perera et al., 2021).

### 4.3 Outcomes on negative conversion time (NCT)/Negative conversion rate (NCR)

NCT and NCR in COVID-19 are strongly related to disease progression and clinical outcomes in patients (Yang et al., 2021) There are only 2 herbs that show good therapeutic results in terms of NCT or NCR outcomes, namely, JY granules and Reduning Injection.

Other herbs showed results on NCT or NCR outcomes that were insignificantly different. However, improved results in NCT and NCR parameters are reported within the treatment group as opposed to the control without statistical significance. In certain clinical trials, a broader array of parameters such as symptom recovery, the mean increase in oxygen saturation (SpO2) level, reduction in the percentage of lung inclusion on CT, and improvement in CRP test results were considered. NCT/NCR parameter did not show a significant difference between the control and test groups.

### 4.4 Strengths and limitations

Herbal medicines have been an important part of traditional medicine practices in the community for centuries. Their use has empirically proven their benefits in overcoming various health problems. However, to increase the trust and validity of using herbal medicines, clinical studies have been conducted to scientifically validate their effectiveness. The summarized results of these clinical trials can be an important reference in developing phytopharmaceuticals, which are medicinal products derived from plant ingredients. Thus, the combination of empirical knowledge and scientific evidence from clinical studies can provide a strong basis for the use of herbal plants as a reliable therapeutic alternative in modern medicine. The strength of this study summarizes all clinical trials on herbal medicines using the randomized controlled trial method with specific outcomes. This review is exclusively a systematic review in nature and lacks statistical analysis, preventing a definitive conclusion regarding which herbs are most effective in terms of LOS, NCT, and NCR outcomes.

In addition, there are several potential biases and study heterogeneity that need to be considered in this systematic review. First, the variability of herbal medicine formulations can be a source of heterogeneity between studies. Various herbal medicines can have different compositions, both in active ingredients and concentrations, which can affect study results (Wang et al., 2023). Second, the method of administration of herbal medicines may also vary between studies, including dosage, frequency of administration, and mode of ingestion (e.g., oral, topical, or inhalation). These differences may affect the rate of absorption, distribution, metabolism, and excretion of herbs in the body, as well as the observed treatment outcomes (Sun et al., 2019). Third, heterogeneity in the patient population, such as differences in age, gender, premenopausal health conditions, history of chronic diseases, and use of other medications, may affect the response to herbal medicines. Studies that include demographically or clinically diverse populations may lead to heterogeneous results that are difficult to synthesize directly. These biases may affect the validity and reliability of the study results and may affect the interpretation and conclusions drawn in the systematic review.

### 4.5 Future studies

Study on herbal medicines related to COVID-19 is conducted as a supplementary therapy and the cases in several countries are currently increasing. The study provides an overview of beneficial herbal medicines with good effects on patients. In the future, a comprehensive systematic review/meta-analysis should be carried out with certain outcomes to compare herbal medicines that provide the best therapeutic results. In addition, the safety aspects of each herbal medicine must be thoroughly analyzed and this consideration becomes crucial in selecting a therapy for COVID-19, specifically in patients with comorbidities.

## 5 Conclusion

In conclusion, valuable insights were provided in the investigation into the efficacy of herbal medicines in patients with COVID-19. The analysis of relevant literature, spanning from 2019 to 2023 through comprehensive searches in prominent databases, focused on key outcomes such as the LOS, NCT, and NCR. The findings contributed to the understanding of the potential benefits of herbal medicines in influencing critical parameters in patients. Herbal medicines worked to treat the pandemic through antiviral, anti-inflammatory, and immunomodulatory mechanisms. These findings can serve as a reference for practical implications that can be considered by healthcare practitioners or polic makers. Healthcare practitioners need to be aware that the use of herbal medicines as adjunctive therapy for COVID-19 may be an option for some patients, especially in countries where the use of herbs has become an integral part of the healthcare system. Therefore, it is important for practitioners to understand the effects, interactions, and appropriate doses of the herbs used, as well as to closely monitor patient responses. Policymakers also need to pay attention to the need for strict regulations related to the use of herbal medicines as adjunctive therapy for COVID-19. Clear and strict regulations will help ensure that the herbal products used are safe, quality, and effective, and limit the risk of misuse or unwanted side effects. However, further review of clinical studies was warranted to establish a strong evidence base and guide the integration of herbal medicines into comprehensive treatment strategies.

## Data Availability

The original contributions presented in the study are included in the article/supplementary material, further inquiries can be directed to the corresponding author.
